# Effects of jasmonic acid signalling on the wheat microbiome differ between body sites

**DOI:** 10.1038/srep41766

**Published:** 2017-01-30

**Authors:** Hongwei Liu, Lilia C. Carvalhais, Peer M. Schenk, Paul G. Dennis

**Affiliations:** 1 School of Agriculture and Food Sciences, The University of Queensland, Brisbane, Queensland 4072, Australia

## Abstract

Jasmonic acid (JA) signalling helps plants to defend themselves against necrotrophic pathogens and herbivorous insects and has been shown to influence the root microbiome of *Arabidopsis thaliana*. In this study, we determined whether JA signalling influences the diversity and functioning of the wheat (*Triticum aestivum*) microbiome and whether these effects are specific to particular parts of the plant. Activation of the JA pathway was achieved via exogenous application of methyl jasmonate and was confirmed by significant increases in the abundance of 10 JA-signalling-related gene transcripts. Phylogenetic marker gene sequencing revealed that JA signalling reduced the diversity and changed the composition of root endophytic but not shoot endophytic or rhizosphere bacterial communities. The total enzymatic activity and substrate utilisation profiles of rhizosphere bacterial communities were not affected by JA signalling. Our findings indicate that the effects of JA signalling on the wheat microbiome are specific to individual plant compartments.

Plants are associated with diverse microbial communities that influence their health and nutrition^1^. These organisms are known collectively as the plant microbiome and could be used to more sustainably maintain or enhance global food security. To achieve this, ways to manipulate the structure of plant-associated microbial communities need to be identified. Recently, activation of the jasmonic acid (JA) plant defence pathway, which is involved in suppression of necrotrophic pathogens and herbivorous insects^2^, was shown to alter the composition of the *Arabidopsis thaliana* root microbiome^3^. Activation of the JA signalling pathway increased the relative abundances of bacterial populations closely related to taxa that are reported to suppress phytopathogens and insects^3^. This suggests that when under attack plants may have evolved mechanisms to recruit symbionts that enhance their tolerance to biotic stress. Currently, however, it is not known whether the microbiomes of other plant species are influenced by activation of the JA pathway, and whether these effects, if any, are also apparent in endophytic compartments of the host.

Given the intimate physical association between plants and endophytic symbionts, changes to the structure of endophytic communities may disproportionately influence host fitness. While JA signalling has been shown to restrict endophytic colonisation of rice (*Oryza sativa*) by incompatible strains of nitrogen-fixing *Azoarcus* bacteria^4^ and suppress nodulation in *Lotus japonicas*^5^, it remains unknown whether JA signalling influences the overall structure of endophytic microbiomes.

Wheat is one of the most important and widely grown crops worldwide. Despite this, the effects of JA signalling on wheat microbial communities have not been characterised. In this study, we used phylogenetic marker gene sequencing to determine whether activation of the JA pathway altered the diversity of bacterial and archaeal communities associated with the wheat rhizosphere and root and shoot endophytic environments. Increased JA signalling was achieved via exogenous application of methyl jasmonate (MeJA) and confirmed by quantification of JA-associated gene transcripts^6^. Lastly, we measured the total enzymatic activity and substrate utilisation profiles of microbial communities associated with the rhizosphere.

## Results and Discussion

### Activation of the JA signalling pathway

**Figure 1:**
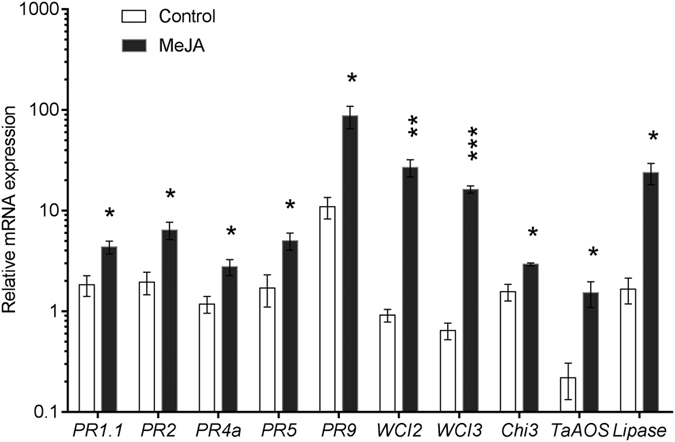
The effect of MeJA application on the transcription of genes associated with the jasmonic acid (JA) signalling pathway in 10-day-old wheat seedlings. Asterisks indicate significant differences between control and MeJA treated plants (*P < 0.05, **P < 0.01, ***P < 0.001, two-tailed student’s *t* test). Error bars represent standard errors of the means (n = 3).

The transcriptional level of ten genes associated with activation of the wheat JA signalling pathway was quantified in shoot tissues 72 hours after MeJA application using real-time PCR (Fig. 1). Previously, we have demonstrated that these genes are strongly associated with the intensity of JA signalling^6^. Relative to the control, MeJA application led to significant increases in the abundance of all gene transcripts as follows: *PR1*.*1* (+2.4 fold), *PR2* (+3.3 fold), *PR4a* (+2.3 fold), *PR5* (+3.0 fold), *PR9* (+8.0 fold), *WCI2* (+29.4 fold), *WCI3* (+25.4 fold), *CHI3* (+1.9 fold), *TaAOS* (+7.0 fold) and *LIPASE* (+14.3 fold) (Fig. 1). These results indicate that the MeJA treatment was successful in activating the JA signalling pathway.

### Root and shoot endophytes

**Figure 2:**
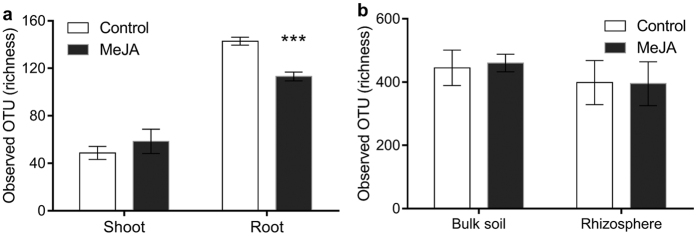
The effect of MeJA treatment on the observed numbers of bacterial taxa (OTUs) associated with (**a**) wheat shoot and root endophytic environments, (**b**) bulk soil and the wheat rhizosphere. The asterisks indicate significant difference (*P* < 0.001) between treatments. All values were based on 1,250 rarefied sequences per sample. Error bars denote standard errors (n = 3).

**Figure 3:**
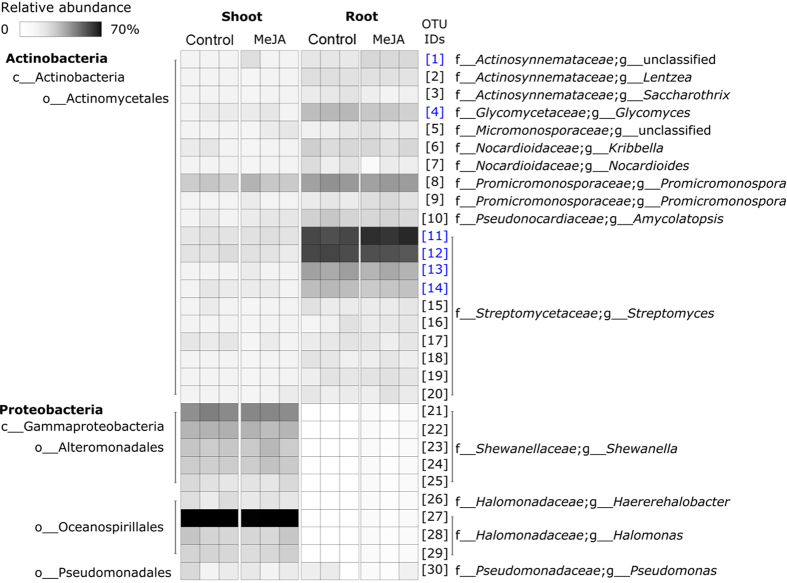
Heatmap summarising variation in the composition of bacterial communities associated with wheat shoot and root endophytic environments with or without MeJA treatment. Each Operational Taxonomic Unit (OTU) has a unique numeric identifier shown in square brackets that is consistent with those shown in other figures.

Relative to shoots, the diversity of root endophytic communities was richer (Sobs and Chao1) and more even (Simpson’s Diversity Index) (*R*^2^ > 83%, *P* < 0.001) (Figs 2 and S1). This is consistent with the fact that root endophytes typically derive from soil^7^ and that shoot endophytes colonise either from root endophytic environments via the vascular tissue or enter via openings on stems and leaves^8^,^9^. The composition of endophytic communities also differed significantly between roots and shoots (*R*^2^ = 88.9%, *P* = 0.002; Figs 3 and S2). Shoot endophytes were positively associated with members of the *Shewanella* (OTU 21–22) and a representative of the *Halomonas* (OTU 27) (Figs 3 and S2). Root endophytes were positively associated with representatives of the *Streptomyces* (OTUs 11–14) and members of the *Actinosynnemataeae* (OTU 1) and *Glycomyces* (OTU 4) (Figs 3 and S2). All of these taxa have previously been detected as endophytes in a wide-range of plant species. For example, representatives of the *Halomonas* have been observed in endophytic root and shoot environments of: *Alopecurus aequalis*^10^, *Typha domingensis*^11^ and *Arthrocnemum macrostachyum*^12^. *Shewanella* spp. have been detected inside potato tubers^13^, rice roots^14^ and baby spinach leaves^15^. Actinobacteria, particularly *Streptomyces* spp., are frequently isolated from endophytic root and shoot environments of maize (*Zea mays* L.)^16^, rice^17^, tomato^18^ and wheat^19^,^20^,^21^,^22^ and members of the *Streptomycetaceae* are key components of endophytic communities in *Arabidopsis thaliana* roots^23^,^24^.

### The influence of JA signalling on the diversity of root and shoot endophytes

**Figure 4:**
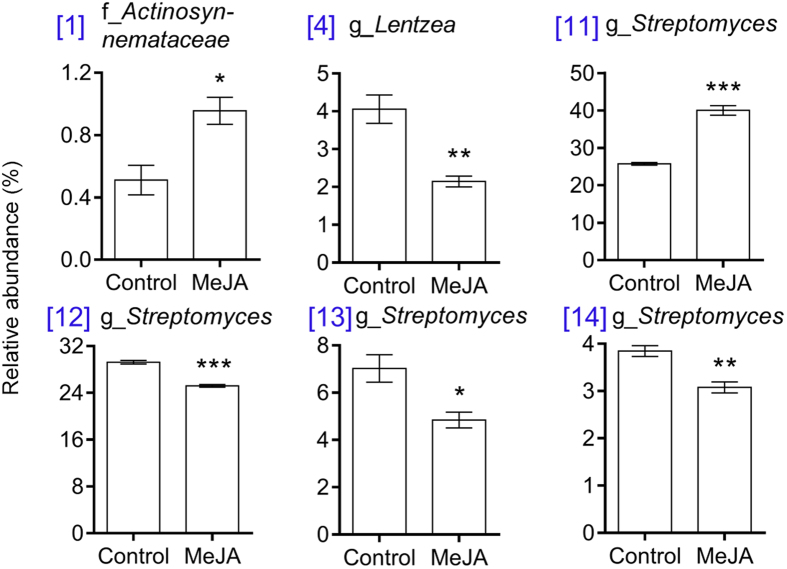
Bacterial Operational Taxonomic Units (OTUs) associated with wheat root endophytic environments that were most strongly affected by MeJA treatment. The asterisks indicate significant differences between treatments (*P < 0.05, **P < 0.01, ***P < 0.001, two-tailed student’s *t* test). Each OTU has a unique numeric identifier shown in square brackets that is consistent with those shown in other figures.

Activation of JA signalling led to a significant reduction in the richness (*P* < 0.001) and evenness (*P* < 0.001) of root, but not shoot, endophytic communities (Figs 2 and S1). This novel finding may indicate that when under attack plants have evolved a mechanism to generally suppress microbial colonisation. However, absolute rather than relative abundances are needed to test this hypothesis. Previous studies have also reported no effects of JA signalling on the diversity of endophytes associated with aerial parts of plants^25^. Root endophytic communities may be more responsive to JA signalling because, relative to aboveground environments, soils harbour more organisms and, therefore, more potential attackers. Activation of JA signalling also led to a significant change in the composition of root, but not shoot, endophytic communities (*P* = 0.011; Figs 3 and 4 and S2). Relative to the control, MeJA treatment significantly increased the relative abundances of a *Actinosynnemataeae* (OTU 1) and a *Streptomyces* (OTU 11) population, and decreased the relative abundances of a *Glycomyces* (OTU 4) population and several members of the *Streptomyces* (OTUs 12–14) (Fig. 4). All of these taxa are members of the Actinobacteria, which include many populations that have been shown to promote plant growth, mobilise nutrients and suppress bacterial, fungal or viral phytopathogens^26^,^27^,^28^,^29^,^30^. For this reason, the observed changes in the relative abundances of actinobacterial populations in our study, may have had functional consequences for the host, which deserve further investigation in future studies.

### Rhizosphere and bulk soil microbial communities

**Figure 5:**
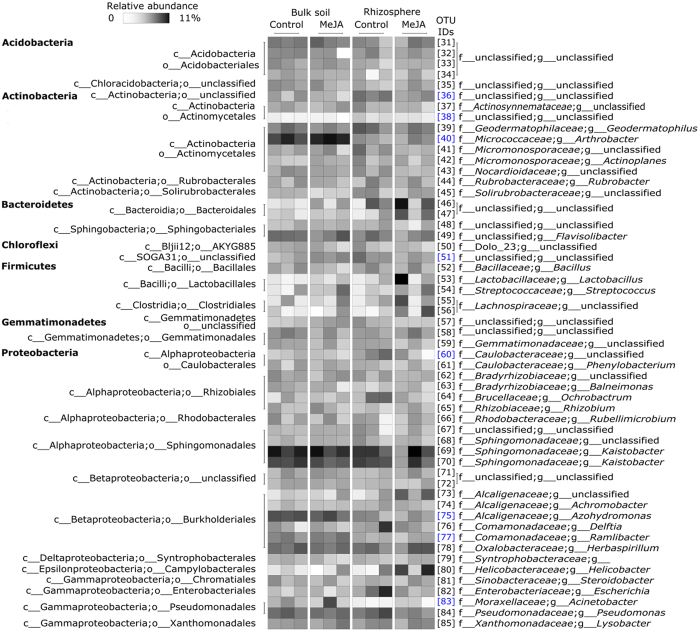
Heatmap summarising variation in the composition of bacterial communities between bulk soil and the wheat root rhizosphere with or without MeJA treatment. Each Operational Taxonomic Unit (OTU) has a unique numeric identifier shown in square brackets that is consistent with those shown in other figures. OTUs highlighted in blue differ between bulk soil and the wheat rhizosphere (*P* < 0.05).

Activation of the JA pathway did not significantly influence the richness, evenness or composition of bacterial communities associated with the rhizosphere or bulk soil (*P* > 0.05) (Figs 2 and 5 and S1). Likewise, activation of the JA pathway did not influence the total enzymatic activity or substrate utilisation profiles of microbial communities associated with rhizosphere or bulk soil (Fig. S3). While all previous studies indicate that JA signalling has no effect on the richness or evenness of rhizosphere bacterial communities^3^,^31^, the effects on bacterial community composition are inconsistent. When grown in soil collected from areas where *A. thaliana* grows naturally, stimulation of the *A. thaliana* JA pathway led to a significant alteration in rhizosphere bacterial community composition^3^. However, when grown in ‘non-native’ soils, induction of the *A. thaliana* JA pathway had no effect on the composition of rhizosphere bacterial communities^31^. This suggests that JA pathway-mediated effects on rhizosphere bacterial communities may be influenced by soil type and the length of association between a particular plant genotype and soil. The soil selected in our study had a long cropping history of wheat but we did not detect any effects on rhizosphere bacterial communities within three days of JA signalling. This does not rule out the possibility that effects may become apparent over longer time periods or for plants grown in other soils.

As observed in many studies^32^,^33^, the composition of bacterial communities in the rhizosphere differed from those of those associated with bulk soil (*R*^2^ = 13.3%, *P* = 0.048; Figs 5 and S4). The rhizosphere was associated with larger relative abundances Actinomycetales (OTU 36, 38), Chloroflexi (OTU 51) and Caulobacteraceae populations (OTU 60), while bulk soil was positively associated with members of *Arthrobacter* (OTU 40), *Azohydromonas* (OTU 75), *Acinebacter* (OTU 83) and *Ramlibacter* (OTU 77) (Figs 5 and S4). Relative to bulk soil, the rhizosphere was also associated with more microbial enzyme activity (*P* < 0.001; Fig. S3). Bacterial community richness and evenness (Figs 1 and S1) and microbial substrate utilisation profiles (Fig. S3), however, were similar between rhizosphere and bulk soil samples.

### Effects of JA signalling on root and shoot biomass

Relative to the controls, MeJA treatment led to a 14% reduction in root dry weight (*P* = 0.015) but shoot biomass was not affected (Fig. S7). This is consistent with previous studies in *Arabidopsis thaliana*^34^,^35^ and sunflower (*Helianthus annuus L*.)^36^, which reported root inhibition upon activation of JA signalling.

## Conclusion

Our study demonstrates that activation of JA signalling in wheat reduces the diversity and changes the composition of bacterial communities in endophytic roots but not in shoots or in the rhizosphere. Most of the root endophytic populations that became more abundant in response to JA signalling were closely related to taxa previously reported to suppress bacterial, fungal or viral phytopathogens, promote plant growth or mobilise nutrients^26^,^27^,^28^,^29^,^30^. JA signalling also led to a decrease in root biomass, which suggests that plants prioritise defence over growth when under attack. We hypothesis that the change in root endophyte communities in response to JA signalling may reflect a coevolved mechanism by which plants recruit microbial symbionts that enhance host biotic stress tolerance when under attack.

## Materials and Methods

### Plant growth conditions and experimental design

Wheat (*Triticum aestivum*) seeds (Crusader variety) were pre-germinated on a moist filter paper in a petri-dish for 36 h and then planted in 30-well punnet trays with three seeds per well (Fig. S5). Plants were grown in soil collected from 0–10 cm depth in a long-term wheat paddock in Condamine, Queensland, Australia (26.90°S, 149.64°E). Key physicochemical characteristics of this soil are summarised in Table S1. The soil was a mesotrophic effervescent Brown Sodosol developed on Cainozoic sand plains and had been under no-till management for 19 years. This paddock has a long cropping history of wheat and the previous crop on this soil was also wheat. The soil contained 25% clay, 14% silt and 61% sand and was homogenised prior to planting using a 2.4 mm sieve. Two additional trays were filled with soil but were not planted (Fig. S5). All trays were transferred to a controlled environment chamber (Percival Scientific, Boone, IA, USA) at 20 °C with a photoperiod of 12 h and light intensity of 150 mmol m^−2^ s^−1^. Throughout the experiment, the plants were watered once per two days with an amount ~10 mL per well, and the positions of the trays within the growth chamber were changed on a daily basis.

After 10 days (two-leaf stage), the JA signalling pathway was activated by exogenously applying methyl jasmonate (MeJA) as previously described^3^. Briefly, 300 μL, 0.5% (v/v ethanol) of MeJA was applied on a cotton ball attached to the lid of the tray to create an atmosphere containing 0.025 μL MeJA L^−1^. The tray was then immediately sealed with tape and enclosed in two sealed transparent plastic bags. The same procedure was repeated for the control plants but MeJA was omitted and 300 μL of ethanol which was the solvent used to prepare MeJA solution was applied to the cotton ball. To determine whether MeJA led to any direct effects on soil microorganisms one of the unplanted trays was treated with 300 μl MeJA solution and compared to another tray that was treated with 300 μl ethanol. We included three replicates per treatment. Each plant replicate comprised a pool of 30 plants.

### Sample collection

#### Bulk soil and rhizosphere samples

All samples were collected 72 h post-MeJA treatment (Fig. S5). For bulk soil samples, soil was collected in sterile tubes and then stored at −80 °C until further processing. For rhizosphere soil samples, roots were carefully removed from each pot, excess soil was removed by shaking and that remaining closely adhered to the roots was considered to be rhizosphere soil^3^. For DNA extraction, rhizosphere soil was recovered by shaking roots in sterile 50 ml tubes each containing 25 ml sterile phosphate buffer (Na_2_HPO_4_ 7.1 g, NaH_2_PO_4_·H_2_O 4.4 g, amended to 820 mL, pH 7.0, 0.1 M) for five min at 250 rpm. After shaking, roots were transferred to new tubes and rhizosphere soil was pelleted by centrifugation at 12,000 g for 3 min then transferred to −80 °C storage until further processing. For MicroResp^TM^ (James Hutton Institute, Invergowrie, Scotland, UK)^37^, rhizosphere soil was physically separated from roots using sterile gloves.

#### Root and shoot endophytic samples

After removal of rhizosphere soil, root tissues were washed with distilled water and 0.1% Silwet L-77 in phosphate buffer three times^38^, sonicated at 20 kHz for five min to remove rhizoplane microorganisms^24^, washed in sterile phosphate buffer, air dried, ground in liquid nitrogen and then stored at −80 °C for DNA extraction. For shoots, half of the tissues were immediately submerged in liquid nitrogen and stored at −80 °C for RNA extraction (Fig. S5). The other half were washed with 0.1% Silwet L-77 in phosphate buffer three times, surface sterilised using 0.5% (v/v) hypochlorite for two min, air dried, ground in liquid nitrogen and then stored at −80 °C for DNA extraction.

#### Determination of plant growth

The MeJA treated and non-treated wheat seedlings were collected 72 h post-treatment and root attached soils were thoroughly removed by washing under distilled water. Thirty plants were pooled in each bioreplicate, and three bioreplicates were included for each treatment. Shoots and roots samples were cut to separate and oven dried (65 °C) for three days, and then the weight of wheat roots and shoots were recorded.

### Quantification of JA signalling pathway-related transcripts

Total RNA was extracted from wheat shoots using the SV Total RNA Isolation Kit (Promega) according to the manufacturer’s recommendations. The cDNA was synthesised by reverse transcription of 1.5 μg of total RNA using the Superscript III kit (Life Technologies) and both random hexamers and oligo dT primers. Quantitative real-time PCR (qRT-PCR) assays were performed on a ViiA™ 7 sequence detection system (Applied Biosystems, USA). Ten JA defence-related genes in wheat, namely *PR1*.*1, PR2, PR4a, PR5, PR9, WCI2, WCI3, CHI3, TaAOS* and *LIPASE* were examined for gene expression in shoots. Primer sequences are shown in Table S2. The wheat 18S rRNA gene was used as an internal reference gene for normalisation. PCR conditions and the relative expression of each target gene was investigated as previously described^6^.

### DNA extraction and 16S rRNA gene amplification and sequencing

For bulk soil and rhizosphere samples, DNA was extracted from two grams of soil using the Power Soil DNA Isolation kit (MO BIO Laboratories, Carlsbad, CA) according to the manufacturer’s recommendations. For root and shoot samples, DNA was extracted from 0.2 g plant tissue using a CTAB method^39^. Extracted DNA was then quantified using a Qubit^TM^ fluorometer with Quant-iT dsDNA BR Assay Kit (Invitrogen) and normalised to 1 ng μL^−1^ and 20 ng μl^−1^ for soil and plant extracts, respectively.

Bacterial 16S rRNA genes were amplified by PCR with 803 F (5′-ATT AGA TAC CCT GGT AGT C-3′) and 1392wR (5′-ACG GGC GGT GWG TRC-3′) for bulk soil and rhizosphere samples. PCR primers pairs of 799 F (5′-AAC MGG ATT AGA TAC CCK G-3′) and 1193 R (5′-ACG TCA TCC CCA CCT TCC-3′) were used for the amplifications of root and shoot endophytic bacteria. The primer pair 799 F and 1193 R spans the hypervariable regions V5-V6-V7 of the 16S rRNA gene and amplifies preferentially archaeal and bacterial DNA and avoids amplification of plant eukaryotic DNA^38^. For the above two primer pairs, B adaptor (5′-CCT ATC CCC TGT GTG CCT TGG CAG TC-3′) was linked to a key (TCAG) and connected to template specific forward primers. An adaptor (3′-CCA TCT CAT CCC TGC GTG TCT CCG AC-5′) was linked to key (TCAG) and sample specific MID, and then was connected to template specific reverse primer. The MID sequence contained a five-base barcode sequence positioned between the primer sequence and the adapter.

Bacterial and archaeal 16S rRNA genes in soil and endophytic roots and shoots were amplified by PCR which was carried out in a 25 μL reaction containing 14.75 μL ultra-pure water, 5.0 μL 5 × phire buffer, 1.25 μL 10 μM dNTPs, 1.25 μL 10 μM forward primer, 1.25 μL 10 μM reverse primer, 0.5 μL phire^®^ hot start II, and 1 μL of DNA template (1 and 20 ng for soil and plant samples, respectively). PCR conditions were 30 s at 98 °C for initial denaturation, 29 cycles of 10 s at 98 °C, 30 s at 56 °C for the annealing step and 45 s at 72 °C, with 7 min of 72 °C for final extension step.

Amplicons of the 16S rRNA gene (~400 bp) generated by PCR primers 799 F and 1193 R were excised from an agrose gel (1.5%) and were further purified using a Wizard^®^ SV Gel and PCR Clean-Up System (Promega). After purification, amplification products were quantified using a Qubit™ fluorometer with Quant-iT dsDNA HS Assay Kits (Invitrogen), normalised to 25 ng μL^−1^ per sample and then pooled for 454 pyrosequencing. Sequencing was performed by Macrogen (Seoul, Korea).

### Processing of sequence data

Data were processed as described previously^40^. Briefly, sequences were quality filtered and dereplicated using the QIIME script split_libraries.py with the homopolymer filter deactivated^41^, checked for chimeras against the GreenGenes database (October 2013 release) using UCHIME ver. 3.0.617^42^, homopolymer error corrected using Acacia^43^ and then subjected to the following procedures using QIIME: (1) OTUs were picked at 97% similarity, (2) OTU representative sequences were assigned GreenGenes (October 2013) taxonomy using BLAST, and then (3) tables with the abundance of different operational taxonomic units (OTUs) and their taxonomic assignments in each sample were generated. The number of reads was rarefied to 1,250 per sample to allow comparisons of diversity without the bias of uneven sampling effort. The mean number of OTUs (observed richness) and Simpson’s Diversity Index values corresponding to 1,250 sequences per sample were calculated using QIIME.

### Microbial community activity

Community-level physiology profiles (CLPPs) were generated by characterising the induced respiratory responses of microorganisms associated with 0.4 g of each soil sample to 20 substrates using MicroResp^TM^ 37 as described in Liu *et al*.^44^. The substrates included carboxylic acids (citric acid, methyl pyruvate, oxalic acid, D+ galacturonic acid and succinic acid), carbohydrates (beta-d-fructose, D-(+)-trehalose, D-glucose, L-malic acid, D-xylose, mannitol, L-(+) Arabinose, cellulose), amino acids (L-alanine, gamma-aminobutyric acid, L-arginine, L-Asparagine), urea, uric acid and tween 40. Milli-Q water was added to controls. Fluorescein diacetate (FDA) hydrolysis assays were used to provide a measure of total microbial enzyme activity and were performed as described by Green *et al*.^45^.

### Statistical analyses

The effect of MeJA treatment on enzyme activities and the richness and equitability of bacterial communities was investigated using ANOVA. Differences in transcript abundances and wheat dry weights were assessed using two tailed *t*-tests. The effects of MeJA treatment on the composition of bacterial communities and on substrate utilisation patterns were investigated using Permutational Multivariate Analysis of Variance (PERMANOVA). PERMANOVA was performed using Hellinger transformed OTU abundances. Differences in the abundances of individual OTUs between treatments were identified using ANOVA with posthoc Tukey’s HSD tests. All analyses were implemented using R (version 2.12.0). Differences in the composition of microbial communities or the utilisation of substrates between samples were visualised using principal component analysis (PCA) and/or heatmaps.

## Additional Information

**Accession codes**: The 16S rRNA amplicon sequences associated with this study have been deposited in the NCBI SRA under accession: PRJNA351276.

**How to cite this article:** Liu, H. *et al*. Effects of jasmonic acid signalling on the wheat microbiome differ between body sites. *Sci. Rep.* **7**, 41766; doi: 10.1038/srep41766 (2017).

**Publisher's note:** Springer Nature remains neutral with regard to jurisdictional claims in published maps and institutional affiliations.

## Supplementary Material

Supplementary Information
