# Supplementary material for: Effects of jasmonic acid signalling on the wheat microbiome differ between body sites
Source: Sci Rep. 2017 Jan 30;7:41766. doi: 10.1038/srep41766 (PMC5278374; doi:10.1038/srep41766)
Supplement: Supplementary Information [file srep41766-s1.pdf]

Supplementary Information:

Effects of jasmonic acid signalling on the wheat microbiome differ between body sites

Hongwei Liu[1], Lilia C. Carvalhais[1], Peer M. Schenk[1], Paul G. Dennis[1*]

[1]*School of Agriculture and Food Sciences, The University of Queensland, Brisbane, Queensland 4072, Australia;*

*Correspondence: p.dennis@uq.edu.au

Contents:

Fig. S1 Effect of MeJA treatment on the predicted bacterial richness and evenness..................2

Fig. S2 Effect of MeJA treatment on the composition of root and shoot endophytes.................3

Fig. S3 Effect of MeJA treatment on microbial activity in the rhizosphere and bulk soil..........4

Fig. S4 PCA of bulk soil and wheat rhizosphere bacterial communities....................................5

Fig. S5 Overview of the experimental design and sampling protocol...6

Fig. S6 Rarefaction curves...7

Fig. S7 Effect of MeJA treatment on wheat growth..8

Table S1 Soil physicochemical characteristics...9

Table S2 Primer sequences for real-time PCR..10

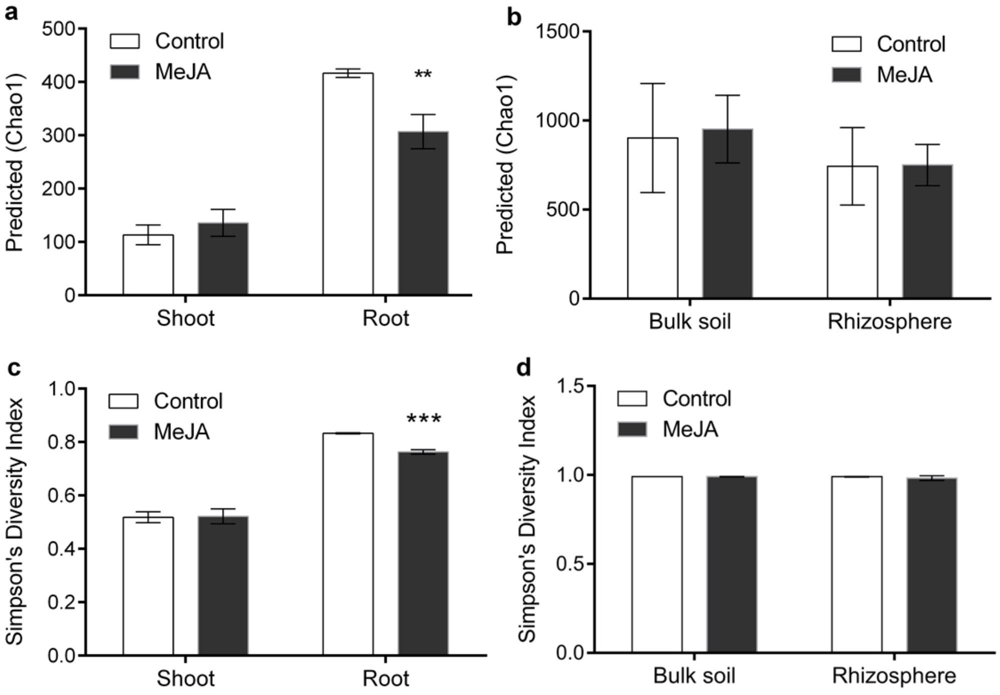

Fig. S1 The effect of MeJA treatment on the (a, b) predicted richness (Chao1) and (c, d) evenness (Simpson's Diversity Index) of bacterial communities associated with (a, c) wheat shoot and root endophytic environments, and (b, d) bulk soil and the wheat rhizosphere. The asterisks indicate significant differences between treatments: $P < 0.01$ $^{(**)}$, $P < 0.001$ $^{(***)}$. All values were based on 1,250 rarefied sequences per sample. Error bars denote standard errors (n = 3).

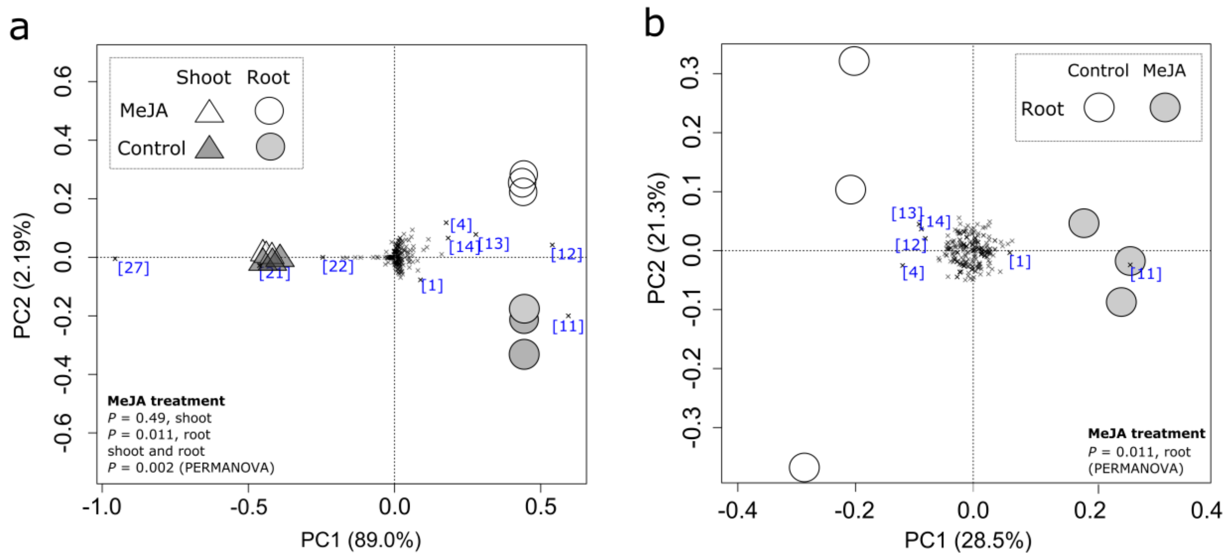

Fig. S2 Principal Component Analysis (PCA) summarising compositional differences in (a) wheat shoot and root endophytic microbial communities; or (b) just wheat endophytic root microbial communities. The numbers in square brackets represent OTU ids and correspond to those shown in Figure 2.

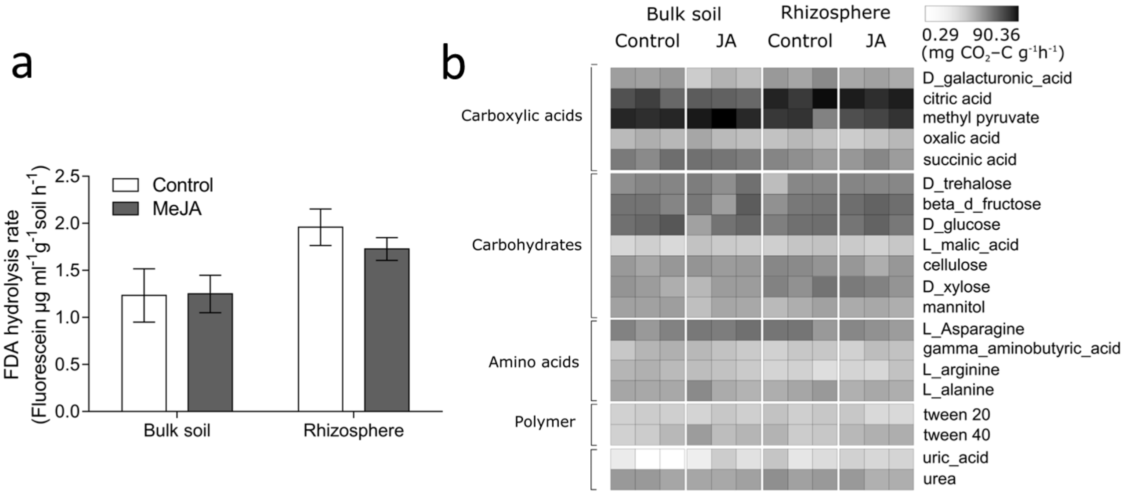

Fig. S3 The effect of MeJA treatment on (a) total microbial enzyme activity as indicated by FDA hydrolysis rates, and (b) substrate utilisation profiles based on Microresp^{TM} assays. Error bars denote the standard errors of the mean (n = 3).

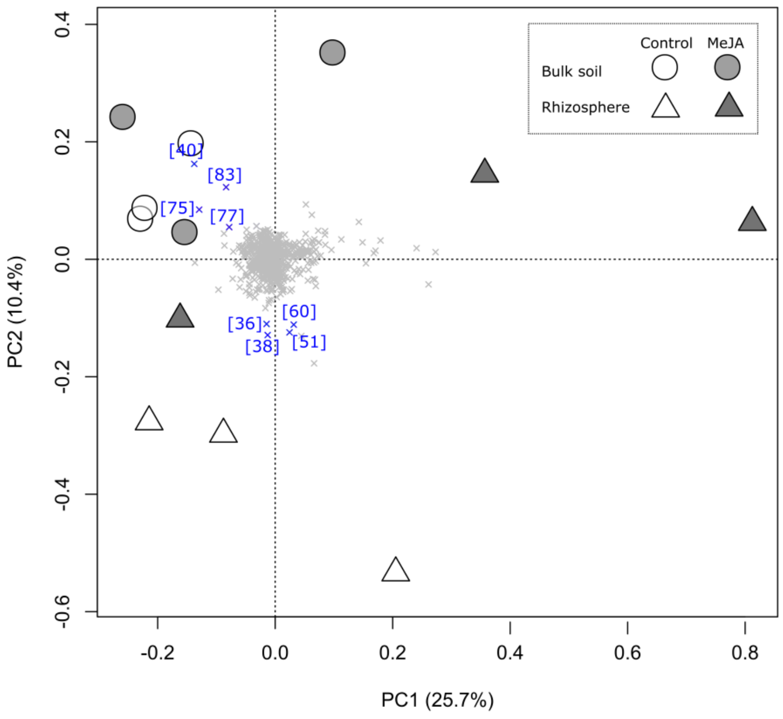

Fig. S4 Principal Component Analysis (PCA) summarising variation in the composition of bacterial communities associated with the wheat rhizosphere and bulk soil environments. The numbers shown in square brackets represent OTU ids and correspond to those shown in Figure 5.

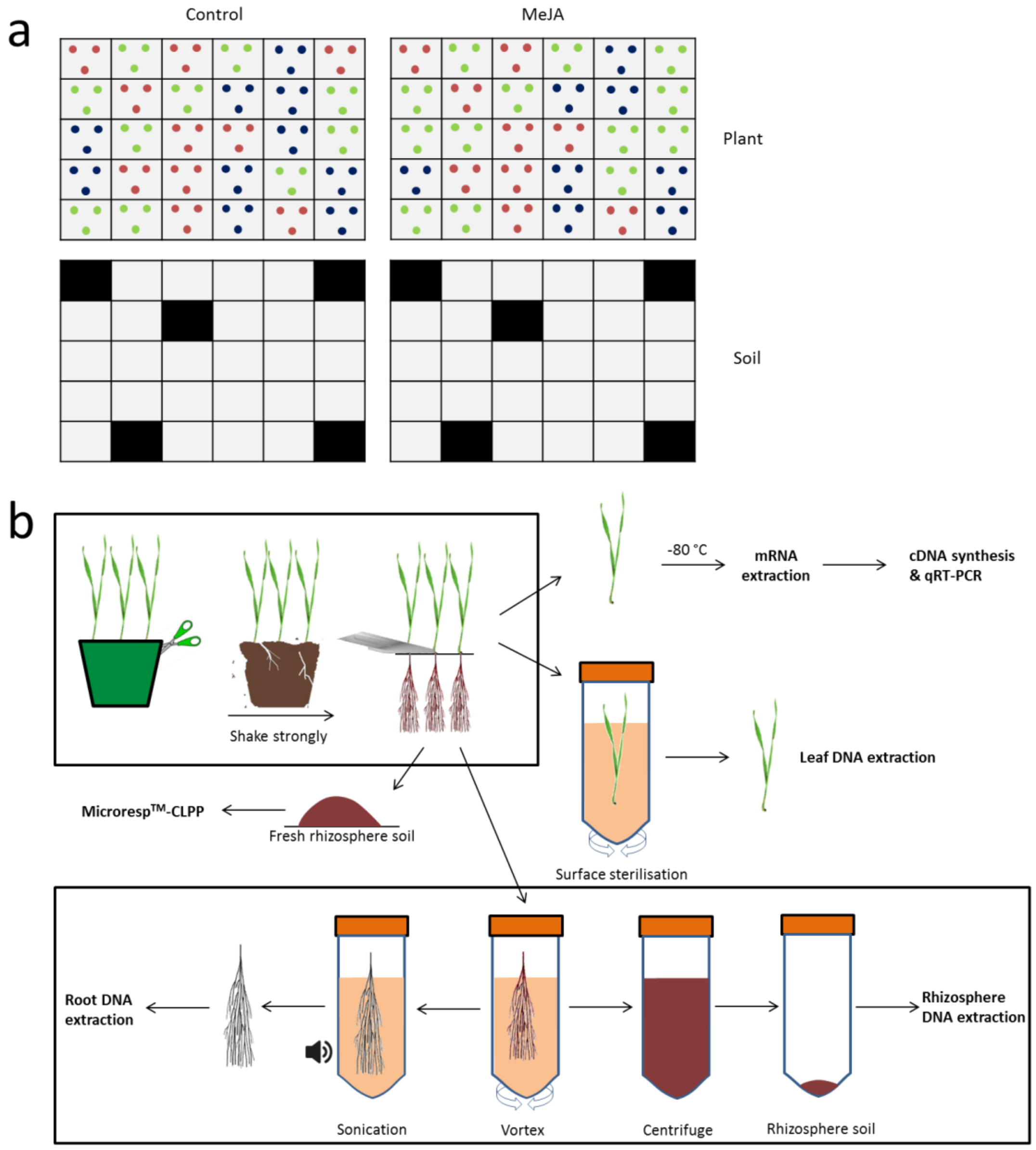

Fig. S5 Experiment design (a) and sampling (b). In panel a the four grids represent punnet trays. Half are used for plants which are represented by red, green and blue dots that indicate different bioreplicates. The other trays were used for bulk soil and were only partly filled as indicated by the black squares. Panel b shows the sampling methods, which correspond to the discriptions in the Materials and Methods of this study. Breifly, rhizosphere soil was collected using the 'pull and shake' method. The sampling procedures for the rhizosphere soil, and the wheat root and shoot tissues as well as surface sterilisation methods are detailed in the Materials and Methods.

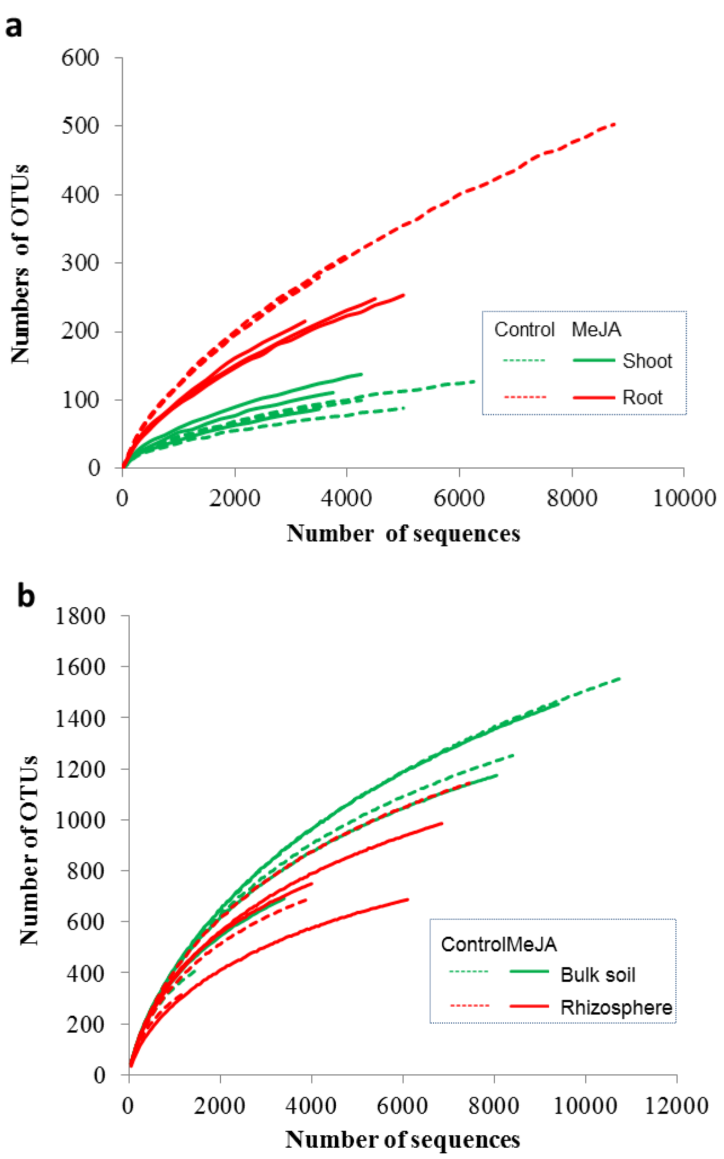

Fig. S6 Rarefaction curves showing that the communities were not exhaustively sampled.

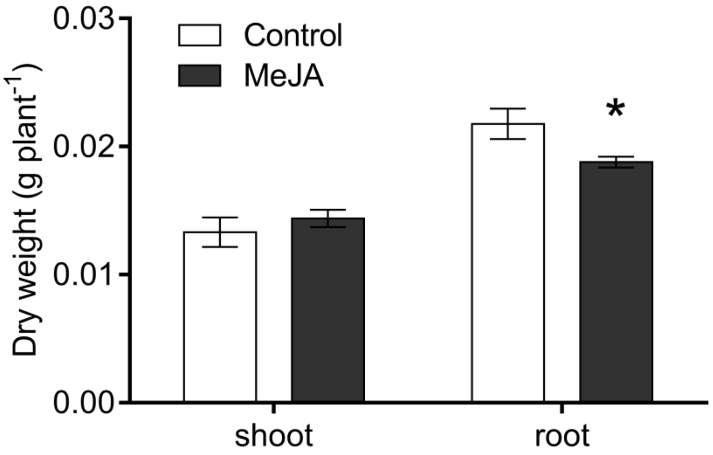

Fig.S7 Bar chart showing the influence of MeJA treatment on dry weight of shoots and roots of 10-day-old wheat seedlings. The asterisk above the column indicated a significant difference between mock and MeJA treatments ($P < 0.05$ (*), two-tailed student's t test). Error bars denote the standard error of the mean (n = 3).

Table S1 Soil physicochemical characteristics

Soil parameter	Value
pH	7.4
Organic carbon content (%)	1.28
Total carbon content (%)	1.36
Total nitrogen (%)	0.18
Total Al concentration (ppm)	30590
Total Ca concentration (ppm)	1781
Total Cu concentration (ppm)	17
Total Fe concentration (ppm)	21013
Total K concentration (ppm)	1666
Total Mg concentration (ppm)	154
Total Mn concentration (ppm)	1936
Total Na concentration (ppm)	933
Total P concentration (ppm)	278
Total S concentration (ppm)	355
Total Zn concentration (ppm)	46

Table S2 Primer sequences used for real-time PCR assays designed to confirm methyl-jasmonate induced activation of JA signalling in wheat.

Accession	Gene	Forward primer sequence	Reverse primer sequence	Gene description
[1]AF159369	*18S*	CAAAGCAAGCCTACGCTCT	ATACGAATGCCCCCGACT	*Haematococcus pluvialis* 18S ribosomal RNA gene
[2]AJ007348	*PR1.1*	CTGGAGCACGAAGCTGCAG	CGAGTGCTGGAGCTTGCAGT	*PR1* (basic), pathogenesis-related protein 1
[2]Y18212	*PR2*	CTCGACATCGGTAACGACCAG	GCGGCGATGTACTTGATGTTC	beta-1,3-endoglucanase
[2]AJ006098	*PR4a*	CGAGGATCGTGGACCAGTG	GTCGACGAACTGGTAGTTGACG	*wheatwin 1-2* gene
[2]AF442967	*PR5*	ACAGCTACGCCAAGGACGAC	CGCGTCCTAATCTAAGGGCAG	WAS3a thaumatin-like protein
[2]X56011	*PR9*	GAGATTCCACAGATGCAAACGAG	GGAGGCCCTTGTTTCTGAATG	wheat peroxidase
[1]AB029936	*CHI3*	GACCTCCTTGGCGTCAGCTA	TGCATGTCTTCTCGCATCATATAGTC	class 1b neutral chitinase
[2]U32428	*WCI2*	TAGGAACTGGAACTTCACCGAGC	GGTAGTCCTTGATGTGCAGCGAC	wheat chemically induced (*WCI*) gene, Lipoxygenase (Fragment)
[2]U32429	*WCI3*	AAAGTTGGTCTTGCCACTGACTG	TCGACAAAGCACTTCTGGATTTC	wheat chemically induced (*WCI*) gene, sulfur-rich/thionin-like protein
[1]AY196004	*TaAOS*	TCCCGAGAGCGCTGTTTAAA	GACGATTGACGGCTGCTATGA	*Triticum aestivum* allene oxide synthase
[3]TaBs117A2	*LIPASE*	CACAAAATATCGACCCACCAC	ACTGGGTATTCGTCTGTCAGC	wheat lipase

[1]Liu, H., Carvalhais, L. C., Kazan, K., Schenk, P. M. Development of marker genes for jasmonic acid signaling in shoots and roots of wheat. *Plant Signal. Behav.* 11(5), e1176654 (2016).

[2]Desmond O. J., Edgar, C. I., Manners, J. M., Maclean, D. J., Schenk, P. M., Kazan, K. Methyl jasmonate induced gene expression in wheat delays symptom development by the crown rot pathogen *Fusarium pseudograminearum*. *Physiol. Mol. Plant P.* 67(3), 171–179 (2006)

[3]Lu, Z., Gaudet, D., Puchalski, B., Despins, T., Frick, M., Laroche, A. Inducers of resistance reduce common bunt infection in wheat seedlings while differentially regulating defence-gene expression. *Physiol. Mol. Plant P.* 67(3), 138-148 (2006).
